# Biopolymers in Drug and Gene Delivery Systems 2.0

**DOI:** 10.3390/ijms242317099

**Published:** 2023-12-04

**Authors:** Yury A. Skorik

**Affiliations:** Institute of Macromolecular Compounds of the Russian Academy of Sciences, Bolshoi VO 31, 199004 St. Petersburg, Russia; yury_skorik@mail.ru

In recent years, significant progress has been made in the design and development of biopolymer-based delivery systems for a wide range of applications, including cancer therapy, gene editing, regenerative medicine, and vaccine delivery. These advances have paved the way for more precise and effective treatments, minimizing off-target effects and reducing the frequency of dosing. In addition, the advent of cutting-edge technologies such as 3D printing and nanotechnology has made it possible to create intricate biopolymer-based delivery systems with tailored properties, enabling precise modulation of drug release kinetics and spatial distribution. This level of customization holds great promise for improving therapeutic outcomes and minimizing side effects.

Importantly, the convergence of biopolymer science with other disciplines, such as molecular biology, materials science, and bioengineering, has led to the development of multifunctional delivery platforms that can overcome biological barriers and deliver therapeutics to specific cellular targets. This convergence has the potential to change the treatment landscape and offers hope for diseases once considered incurable. As we face the challenges of drug resistance, personalized medicine, and the demand for safer and more effective therapeutics, the role of biopolymer-based drug and gene delivery systems becomes increasingly important. The ability to tailor delivery systems to individual patient profiles, genetic variations, and disease characteristics represents a paradigm shift in healthcare that brings us closer to the realization of precision medicine.

The development of biopolymeric drug and gene delivery systems holds great promise for the future of medicine. The following are some potential future prospects, which are discussed further in recent reviews on the subject:

- Targeted and personalized medicine. Biopolymeric delivery systems have the potential to enable the targeted and personalized delivery of drugs and genes to specific cells or tissues in the body. This could lead to more effective and efficient treatment of various diseases with fewer side effects [1,2,3].
- Improved stability and bioavailability. Future research and development efforts in biopolymeric drug delivery systems could focus on improving the stability and bioavailability of drugs, particularly those with poor solubility or limited absorption. This could lead to more effective and reliable therapies for a wide range of diseases [4,5].
- Integration of smart materials and technologies. Future developments in biopolymeric delivery systems may include the integration of smart materials and technologies, such as stimuli-responsive polymers [6], nanotechnologies [4,7], plasma technologies [8], cryogenic technologies [9,10], 3D printing [11,12], and electrospinning [13,14], to enable better control of drug release and distribution in the body. This could lead to more precise and predictable therapeutic outcomes.
- Combination therapies. Biopolymeric delivery systems could facilitate the development of combination therapies in which multiple drugs or genes are delivered simultaneously to target different aspects of a disease or to treat multiple coexisting conditions. This could lead to synergistic effects and improved patient outcomes [15,16].
- Non-invasive and sustained delivery. Future biopolymeric delivery systems may be designed to enable non-invasive and long-lasting delivery of drugs, potentially reducing the need for frequent dosing and improving patient compliance with treatment regimens [17,18].
- Regulatory and commercialization challenges. As biopolymeric delivery systems continue to advance, there will be a need to address regulatory and commercialization challenges, such as standardizing manufacturing processes, ensuring safety and efficacy, and addressing intellectual property issues [19].

Overall, the future of biopolymeric drug and gene delivery systems is bright and has the potential to revolutionize the way we deliver and administer therapeutic agents, leading to improved patient outcomes and a more personalized approach to medicine. It is therefore with great pleasure that we introduce the Special Issue “Biopolymers for Drug and Gene Delivery Systems 2.0”. Building on the success of the first volume of this Special Issue [20], the second volume continues to explore the exciting developments in the field of biopolymer-based drug and gene delivery systems. In the ever-evolving landscape of pharmaceutical research and development, biopolymers have emerged as a promising class of materials for delivering therapeutics with enhanced precision and efficacy. The first volume of this Special Issue [20] provided some interesting examples of recent research and developments in the design, synthesis, and application of biopolymers for drug and gene delivery, highlighting their unique advantages, such as biocompatibility, biodegradability, and low immunogenicity. It highlighted the potential of biocompatible and biodegradable polymers to overcome the challenges associated with conventional drug and gene delivery systems, offering solutions such as targeted delivery, sustained release, and enhanced therapeutic efficacy.

In this second volume, we are proud to present a collection of eight original research articles and one review that delve deeper into the innovative applications, cutting-edge technologies, and emerging trends in the field of biopolymer-based drug and gene delivery systems. The contributors to the Special Issue “Biopolymers for Drug and Gene Delivery Systems 2.0” come from different countries, including Russia, the UK, France, Greece, and Japan, and the papers cover a wide range of topics, including the design and synthesis of novel biopolymer-based delivery carriers, the optimization of delivery strategies for specific therapeutics, and the exploration of advanced targeting and controlled release mechanisms. We are confident that the valuable insights and findings presented in this Special Issue will inspire and guide future research efforts in the exciting field of biopolymer-based drug and gene delivery. We hope that the articles presented in this Special Issue will serve as a source of inspiration and knowledge for researchers and practitioners, contributing to the continued advancement of biopolymer-based drug and gene delivery systems. We look forward to seeing the impact and implications of these advances in the translation of innovative therapeutic solutions for the benefit of patients and society.
